# The New York State Veterinary Medical Society

**Published:** 1893-02

**Authors:** N. P. Hinkley

**Affiliations:** Secretary


*The New York State Veterinary Medical Society.*—The New York State Veterinary Medical Society, organized January 15th, 1890, and incorporated March 17th, 1891, held its third annual meeting on Thursday, January 12th, 1893, at ten o'clock A. M., at the Assembly Room of the Vanderbilt House, Syracuse, N. Y.

The meeting was called to order at ten o'clock A. M., by Dr. Wende, the Vice-President, who called upon the Secretary to read the roll.

The following members answered: Drs. John A. Bell, Watertown; W. L. Baker, Cortland; James Carnrite, Amsterdam; Chas. Cowie, Ogdensburg; J. M. Chase, Seneca Falls; W. H. Carpenter, Johnstown; A. Drinkwater, Rochester; O. B. French, Honeoye Falls; Wilson Huff, Rome; N. P. Hinkley, Buffalo; E. D. Hayden, Syracuse; R. S. Huidekoper, New York; Prof. James Law, Ithaca; Drs. H. Sutterby, Batavia; John Wende, Buffalo; W. E. Willyoung, Albion; V. L. James, Cooperstown; J. A. Genung, Ithaca; James A. Pendergast, Phoenix; G. G. Dean, Tully; D. H. Rowe, Little Falls; D. C. Papworth, Baldwinsville.

Dr. Wende:—Gentlemen: With great pleasure I introduce to the Society Dr. Huidekoper, of New York, the President, who unfortunately was unable to be with us last year.

Dr. Huidekoper then assumed the chair and said: The Secretary will read the minutes of the last meeting. On motion the reading of the minutes was dispensed with. The President then gave a short address as follows:

Gentlemen:—I have to say a few words only of greeting as President of Society, and of explanation and apology as an individual.

When one year ago I received a telegram stating that I had been elected President of this Association, toward which I had done practically nothing, the surprise and bewilderment caused by the notice were mixed with curiosity as to its authenticity, and I hesitated about sending any reply, until one of your members called upon me and a letter from your officers confirmed the information.

This unlooked-for honor I could only attribute to certain work that I had done in the past in other associations, and while I felt that my recent adoption of New York State as my residence, and my unavoidable absence from previous meetings of this Association did not render me even eligible for the position, yet as you have seen fit to appoint me, I could do nothing but recognize your courtesy and accept. I wrote to your Vice-President and Secretary, and, asking for their guidance, with the best intentions in the world I resolved to increase the little knowledge which I had of the local veterinary affairs of the State, and to devote as much energy as possible to them. But we all know the various old adages about good resolutions and the failure in their execution.

The busy season of Spring practice in New York City, and other occupations, prevented me from doing more than to keep up a desultory correspondence with your Secretary, until July, when, just as I had really commenced active work to prepare for the subjects which we had to consider at the Summer meeting, I had gone to the country over a day for an operation, and received a telegram which ordered me to Homestead, the seat of the labor riots near Pittsburg, in my capacity as Division Surgeon of the Pennsylvania National Guard. My work there was excessive and continuous, and all other matters were driven out of my thoughts, until noticing one afternoon the date of the month, I remembered it as that of our Association meeting, when I sent a telegram of explanation, which, however, our Secretary tells me he failed to receive On my return accumulated affairs of various kinds forced me to neglect others of our own, but I had the satisfaction of knowing, at least, that they were well looked after by Dr Hinkley, and I really come here as a novice to learn what you have all accomplished, and not as the mentor, which should be the position of one who has merited the honorable position of the presiding chair in this body.

You have before you copies of the programme, which shows the work which we have before us, and it certainly looks formidable to handle in the short period of a a one day's session In the order of business a large portion is routine matter, the execution of which is perfunctory, and the consideration of which has already been attended to by your officers and committees ; but in the work which has been prepared by the latter, there is much which should be received with attention, weighed with impartiality, and acted upon honestly, according to the best judgment of the individual member who casts a vote. It is the duty of the State societies to recognize the importance of the County societies; they should stimulate their members. The State Society should formulate a general law. I offer that to you for consideration ; a report may come from the Secretary.

With the coming year there are one or two important matters to consider. The United States Veterinary Association will hold its annual meeting at Chicago. It is possible its members will want to stop and see a portion of New York State A committee of arrangements should be given the power to take care of them.

Some action should be taken to allow the State Association to change its date of meeting. Visitors to the International Veterinary Congress will probably have a reception in New York City, and the Pennsylvania Society will arrange for a reception in its State. It would be well if this Society would hold its meeting in Buffalo or at Niagara, and receive foreigners on their way to Chicago. All societies should act in harmony.

The next order of business is receiving applications for membership. No new applications were made.

The President: The next order of business is examination of applications by the Censors. Dr. Bell: Chairman, we report the following applications, five of them have been examined and found creditable: W. B. Crandall, of Syracuse, N. Y. (references, Drs. E. R. Havens, and J. A. Pendergast) ; A. Dalton, of Buffalo, N. Y. (references, Drs. Hinkley and Wende) : Harry B. Hanson, (reference, A. Liautard); David S. Kohler (reference, J. K. Sutterby); Thomas Giffen, (reference, Dr. Huidekoper).

The application of T. F. Moadinger was without reference, and was referred to the next meeting.

The President: You have heard the applications. That of Mr. Moadinger will be laid over. As to the others, you have heard the names and the references. What will be your action?

Dr. Baker: I move they be accepted. Mr. President. Dr. Huff: I second the motion. The President put the motion, calling for the yeas and nays, and it was declared carried. The President: The next order of business is introduction of new members. Are any present? Dr. W. B. Crandall arose and said that he duly appreciated the honor conferred when he was received by all members arising.

The President: The next order of business is the report of the Secretary. The Secretary reported as follows:

SECRETARY'S REPORT.

*Mr. President and Gentlemen:*—As secretary of this Society I take pleasure in submitting my annual report for 1892, for your consideration.

In doing so I shall endeavor to give you a true and correct history of the workings, success and present condition of this Society. In fulfilling the duties of my office as secretary I have, during the time intervening since my last report, observed several things which in my opinion benefit us both as a body of professional men as well as individuals to investigate into the causes and to act upon them. I have embodied them in this report and trust you will all become interested in them to discuss and either adopt or dispose of them. And I sincerely trust that no member will think that any remarks or suggestions I may make have been actuated by any personal ambition or desire for gain, as the good and welfare of this Society has always been and always will be with me the first and only consideration either in fulfilment of my official duties, or at its meetings

For the sake of our new members I will briefly state that this Society was first called together and organized by our ex-president, Claude E. Morris, of Bath, N. Y., now located in Brooklyn. It was an earnest appeal to veterinarians in the State to meet at Rochester, N. Y., to formulate ourselves into a State Society for our mutual protection and benefit.

This was in the month of January, 1890, making our birthday January 15th, 1890. About twenty veterinarians were present, and although few in number, they did an excellent day's work for the future of the New York Veterinary Surgeons, by organizing a veterinary society for the Empire State, from which sprang the now flourishing, successful and prosperous New York State Veterinary Society. Our meetings have been held semi-annually in this City, with the exception of the meeting held in New York City, in the month of August, 1891; this was done to induce other Eastern brethren to join us, and to co-operate with us in our endeavors to protect our profession from the many abuses that have been heaped upon us year after year, not only by the quack and unthinking public, but our representatives in the Legislature, and also to unite ourselves into a closer band of friendship and sociability.

To do this we fully realize that we must not be an Eastern, Western, Northern or Southern Society, but as our name implies, a State Society. Hence our reason for meeting in New York, to get New York and Brooklyn veterinarians to join us

I am sorry to add that we were disappointed as regards the number added to our membership, but again, we felt as if our journey had not been in vain, as we were amply repaid by the strong addition of some of New York's and Brooklyn's brightest and most prominent veterinarians to our ranks, who by encouraging words and generous help, gave us every hope for the future of our Society.

We have since then had the pleasure of receiving several applications from all parts of the State, and our Society to-day, its third anniversary, can feel proud of its short, prosperous life.

To-day we number about eighty members in good standing, besides several applications which are now in the hands of the board of censors to be acted upon, and if I am not mistaken, I think we can, with pardonable pride, boast of the largest, most prosperous State Veterinary Society in the Union.

This fact alone should give us fresh vigor to push onward in our good work, until we have every veterinarian of reputable standing in this State a member of our Society ; when we reach this pleasant state of affairs. then, and not until then, can we expect to get just protection by our State laws, for the regulation of veterinary practice.

I find in my position as your Secretary, that a large number of veterinarians in reply to my invitations to our meetings, will write me letters of encouragement and good wishes, but will close with the time-worn excuse, "Am sorry, but will not be able to be with you this time."

Now gentlemen, no doubt but there are times when some of us cannot get away, but I can safely say without fear of contradiction, that nine-tenths of these excuses are without good cause or reason. They all want to see the Society prosper, they all want Legislative protection, but for some reason (the only one I can find) —careless indifference—they fail to come forward and aid us in our good cause.

I sincerely hope that in the near future, these luke-warm professional gentlemen will forego some of their vacations for pleasure, or profit, and let us have their company and the honor of their names on our roll. Surely, gentlemen, you all have every reason to be proud of the veterinary profession in the Empire State. We have two veterinary colleges of the highest standing in this State, whose professors are giants in teaching the art.

Of Veterinary Journals, we have the proud distinction of having the only two journals printed in America, pertaining to veterinary medicine and surgery, edited and conducted by veterinarians in New York. Let me here acknowledge myself deeply indebted for the many acts of kindness and courtesy, encouragement and liberality of Prof. Liautard, of the *Veterinary Review*, and Prof. Huidekoper, our worthy President, of the JOURNAL OF COMPARATIVE MEDICINE AND VETERINARY ARCHIVES, who have always opened the pages of their excellent journals for the uses of our Society.

Prof. Liautard not only published our proceedings, but printed the proceedings of our first annual meeting in an extra edition, and sent me five hundred copies for distribution, and would accept no compensation for the same.

These acts of generous kindness should not be forgotten by us as veterinarians, and I think you will all join with me in voting a resolution of thanks to these two valuable journals, and we should not only support them by being a subscriber, but being of assistance to them in sending records of cases of interest and original articles in veterinary medicine and surgery. We have the material to do so ; it is only the same excuse—"careless indifference"—willing to be benefited, but not willing to benefit others. In the past year new colleges, teaching the veterinary art, have been incorporated in different States, and I am sorry to say that we, as veterinarians, cannot honorably sanction their published curriculum. I trust that this society will take such action at this meeting as will show its dislike and feelings in the traducing of our profession.

Since my last report, I have the gratifying news to tell you that our Bureau of Animal Industry, by its constant watching and good work, has been able to proclaim the States now free from that terrible scourge pleuro-pneumonia. I say all praise to this able body of veterinarians who have placed our American meats on all European markets, by stamping out this terrible disease. The same Bureau has also formulated a complete chain of veterinary inspection of all live-stock coming into this country from the Dominion of Canada. It has also, at the request of several large pork and beef packing houses, placed competent veterinarians and microscopists for the sanitary inspection of all meats for exportation and home consumption And although there is room for improvement in the present system of inspection of meats, live-stock and food products, and the numerous herds of cattle used for producing milk for public consumption, which we hope our chief veterinarians at Washington will in time be able to remedy, we must also be thankful for the rapid advance made in the past few years.

The increasing interest taken by our horsemen, and the fabulous amount of capital invested in the importing and breeding of thoroughbred and American trotting horses, should cause every veterinarian to redouble his energies to keep well posted on breeding and its many serious and complicated diseases. He should let the stockmen know and feel that the educated veterinarian is his guide and pilot to direct him through epidemics of serious nature, and to steer him clear of rocks and shoals of ignorance, charlatans, patent nostrums and quacks.

Since my last report, I have received several letters from veterinarians of different parts of the State, saying that there were several counties where the present law pertaining to the regulation of veterinarian practice was being violated, and that the District Attorneys refused to prosecute, asking me for aid, which, of course, in our present condition, I was unable to give them.

I respectfully suggest that you, as a society, will take action in this matter to-day, and authorize a committee of one or more to act as a prosecuting committee, with power to act in procuring witnesses, and the society pledge itself to pay the expenses of the same. I have also had the important information from some of our veterinarians, of their being placed in the position as defendants in cases of malpractice, through the malice and folly of some rival. We cannot too strongly condemn this as veterinarians, and must stand together in cases of this kind. In addition to sending our aid, not only to prosecute, we should give them every and any honorable assistance in time of need.

I suggest that a clause be added to our code of ethics to this effect. I also suggest that a committee on Legislation take advice from some competent lawyer as to the probability of getting a law similar to that of the medical fraternity, throwing out the testimony of non-graduates as incompetent.

I would also like to call your attention to the next meeting of the United States Veterinary Medical Society, which will be held in Chicago next September, during the World's Fair; and I, as a member, would like very much to see every member of our Society not only attend, but become a member of this excellent and instructive association, as I believe it is intended to make it an international congress of veterinarians. It will bring a great many European veterinarians into our midst, giving us an opportunity to become more clearly acquainted with them, an opportunity none of us should lose sight of. In my last report I suggested the appointment of County Secretaries. It seems to have met with the general approval of your honorable body, but for some reason they have not been appointed, and I again offer this suggestion.

During the past year I have had printed and mailed 1.000 invitations, 1,000 programmes, 1,000 lists of veterinary surgeons, 500 applications.

Last month I mailed 500 each of invitations, programmes, lists and notices. So far there has only been 17 returned, making a total of 483 having reached their destination. I have revised the lists of veterinary surgeons in this State, now numbering over 500, for the use of my successor, who, I hope, will continue from year to year to keep up this practice, so we will always have a reliable veterinary directory to refer to.

I have received some 200 letters of inquiry, which I have answered. I have also written nearly 300 personal letters to veterinary surgeons, trying to interest them in our works.

Our financial condition will be reported by our Treasurer, Dr. Sutterby I will state, as Secretary, I have not had the desired returns of dues and assessments from some of our members, although frequently reminding them of their little indebtedness, and I trust they will see the necessity of responding more promptly when notified in the future of their indebtedness

The vouchers for expense incurred in my office for the past year are in the hands of the President for your examination and consideration. I have endeavored to be as economical as possible, but should any member feel as though an explanation were necessary, I will be only too glad to give him any information on the subject asked for.

In bringing my report to a close, I again wish to express my thanks to the officers and members of this Society, and to the veterinary journals for their personal aid and generous support, for which I shall ever feel very grateful.

Fraternally yours, N. P. HINKLEY, *Secretary.*

The report was, on motion, accepted and adopted.

The President: You have heard the report of the Secretary, gentlemen ; what is your pleasure in regard to it? Dr. Wende : I move it be accepted. Dr. Bell : I second the motion. The President put the motion and it was unanimously carried.

The President: The next order of business is unfinished business. Secretary Hinkley : I have a report and resolution to appoint a veterinarian in each county, to report anything of interest to this Society. It met with the approval of all the members; but, for some reason no action was taken thereon. The President: Are there any propositions to make ? Dr. Wende: I move that the President appoint the County Secretaries. On motion, seconded and carried.

The President: Reports of Committees are in order. The Committee on Constitution and By-laws reported.

The President: The next is the report of the Committee on Legislation. The Chairman of the Committee reported as follows: We have to report the following draft of a bill to be sent to the Assembly, as follows:

After July 1st, 1893, it shall be unlawful for any person, who is not a graduate of a reputable Veterinary College, to use the title of Veterinary Physician, Veterinary Doctor, Veterinary Dentist, Veterinary Surgeon, or Veterinarian, or any analogous title, for the purpose of professional practice within the State of New York.

Any person not a graduate of a reputable Veterinary College, now in the practice of medicine or surgery on the lower animals in the State of New York, under Chapter . . . of the Laws of New York, of 18 etc., shall, in continuing such practice, do so only under the title of Farrier, and it shall be the duty of the County Clerk of each County to enter in his County Register the word Farrier opposite the name of such practitioner.

Any person violating this act shall be found guilty of a misdemeanor, and it shall be the duty of the District Attorney of the County in which he practices, to prosecute him in the District Court for the same.

Any previou act incompatible with this is hereby repealed.

W. L. Baker,
N. P. Hinkley,
James Law.

Dr. Hinkley: We will have to get the Committee together again. We could not do as much as we desired. We will try to have a satisfactory bill presented at this meeting.

Dr Baker. I would like to have the members meet this afternoon Delay was the trouble last year. I move that it be looked to now. Dr. Bell: I second the motion.

The President: The question will come up this afternoon. The next order is the report of Board of Censors.

The Committee of the Board of Censors reported: That William Somerville, Sr., and Robert M. Somerville, having failed to comply with the request from the Secretary to appear before the Board of Censors at this annual meeting, held January 12th, 1893, and make a proper defense of the charges preferred against them of manufacturing, advertising and selling proprietary medicines, are hereby expelled from membership in the New York State Veterinary Medical Society, and that the Secretary notify them that we demand the return of their certificates of membership in said Society to the Chairman of the Board of Censors.

John A. Bell,
J. M. Chase,
John Wende,
O. B. French,
V. L. James.

The President: The next order is report of County Secretaries. No reports were made.

The President: The next order of business is report of Committee on Publications. Chairman Hinkley: We have but a meagre report, and ask to be continued. Request granted.

The President: The next order of business is report of Committee on Arrangements. Chairman Hinkley reported that arrangements were to have been made for a stenographer and a reception at the hotel. On the Committee were Drs. Hayden and Henderson. I received no reply from them. I telegraphed them twice, and I would suggest that hereafter the President receive the acceptance of Committeemen to act.

The President: The next in the order of business is new business. Are there any subjects to be brought before the Society? It seems there is not. The Secretary will then read the communications and correspondence.

The Secretary: I have a communication from H E. Rowle, of Minneapolis, Minn., a resignation, as he has removed from the State. Dr. Huff: I move it be accepted, with the respect and expressions of good will of the Society. On motion, seconded, so ordered.

The Secretary: The next is the resignation of Dr. D. Leary. Dr Chase (seconded): I move it be acted on, with expression of regret of the Society. Motion seconded, and so ordered.

The President: The next order of business is essays and papers. Is any one ready with a paper on any subject?

Dr. Carnrite: I have a paper—merely a report of a case of a few cows that came under my observation. The owner said he had some cows that didn't seem to be doing just right. There was some swelling in the maxillary glands, and of one hind leg. The animals had had diarrhœa The secretion of milk was suspended. I sounded the lungs, and found them normal.

Dr. Bell: What course did you advise the people to take to stamp out the disease?

Dr Carnrite: He asked me whether or not there was any law regarding the disease of his animals. I told him I would make a report and draw the attention of the Society to the matter. I don't want to make a great spread in this matter. I find people denouncing any ideas that are new. If you advance ideas not in accordance with theirs, you are at once denounced. All the owner wanted was a "little time." I think there is scarcely a herd of cattle in that vicinity that is not affected; tuberculosis prevails to an alarming extent. I have good grounds for such a report. I thought only I would wait and see what was the feeling of the meeting.

The President: What sort of cattle are those in your county?

Dr. Carnrite: A good grade of cattle. I spoke to one or two physicians about the matter, and one said he would report, but he did not. One man said that he had had numerous cases during the winter.

Prof. Law: It may be well to state one fact, $5,000 was appropriated for the State. As I read it, that was to cover the entire expenses. I think, though, we can draw further from the State fund.

The State Board of Health has been empowered to go into the matter, and they are preparing to go before the Legislature at once. The protecting of cattle should be in the hands of the Veterinary Association, not in Boards of Health. First the local health officer should be applied to in cases of tuberculosis, at this time.

He is an associate of the State Board, he notifies them, and they instruct him what shall be done. They will direct him and examine the cases The proper legal course is to hand it to the local Health Board. I think a very good case could be got up by having the matter attended to by properly educated men. A number of cases have come under my observation,—the animals are all right during clear weather, in summer, but when they get out of the bushes, then begins the trouble.

We want to let the people know that the inspection *can be made* satisfactorily to all; but we must have educated men to do it. A few years ago the general public knew nothing of tuberculosis. Now they are better informed. It is a subject of great practical importance to-day. And we should bring public influence to bear on the Legislative body.

The President: Does the law provide that the report shall be made to the local Health Board? Prof. Law: You must apply to the State Board; the local officer is theirs, and entitled to pay. The President: Do you know what pay they get? Dr. Baker: There is no set pay.

The President: I think it is very important to know about reporting tuberculosis. If the law says it shall be done, it *must* be done, but as I read it I do not so understand it. No European countries have so imposed it. In the case of certain diseases, in France, anthrax or glanders for instance, the farmer is held responsible. In Austria and Hungary, a reward is given to those who report cases. Now we know more about tuberculosis than we did some years ago. Theoretically we know it to be contagious, but, practically, we know it requires a predisposing condition in the animal. We owe a duty to the people: also, a duty to ourselves, but we owe a greater duty to the farmer who pays us the money.

Where a dangerous disease *absolutely exists*, as in glanders, it is our duty to report it. I think the reporting of local cases of tuberculosis does an immense amount of damage to the cattle man and the dairy farmer.

Dr. Carnrite: If children are inoculated with the disease, then they die. I would like to say that it almost seems unsafe for a young practitioner to say anything about diseases of cattle. He would be almost afraid to say whether cows were diseased or not. I think if we see anything wrong we should examine, or, if we do not feel competent, have somebody competent do so. If we do not do so, and let people know that there is danger lurking in the meat and milk, we will not be doing our duty.

Prof. Law: I had two cases a year ago. The owner had the reputation of having the best of milk. One cow had a little cough. I went over her very carefully. I thought the case should not be ignored, and gave her a little dose. The next morning the temperature was taken and it was 102, and next 105, and then 106. But I had no hesitation in saying that there was no tuberculosis. On the other hand it is not uncommon to find the bag hard and knotty—but that does not prove tuberculosis. I met a case recently in which the animal was in fine condition, but showed tuberculosis in the lungs and glands They do not dry up; often give more milk. I can heartily endorse what the President has said.

Inoculation is not absolutely protective; it is relative, not absolute. I think, therefore, that susceptibility does not count for so much. I can recall a number of instances in common cattle where I found great susceptibility, until the herd was wiped out. I think it is largely a question of exposure. Of course we should stand on our dignity. We can stand aside and refuse to have anything to do with the matter. I think I am correct in saying that I do not think we are under any obligations to the Board of Health. It is the duty of the public officials to attend to the matter, and it cannot be reasonably expected that we will attend to it.

There are two great ways whereby this disease (tuberculosis) may be transmitted—by the milk and by the meat. How are you to deter it? I suppose many of you like "rare roast beef." Tubercles are not destroyed under 158 degrees. Yet nine out of ten people at the hotels want "rare roast beef." In regard to the competency of the inspectors, they should be veterinarians. I have seen a number of cases where, previous to the death of the animal, one could not correctly diagnose it.

Dr. Lillico: With reference to glanders, I do not know whether any of you have marked the similarity between the bacilli of glanders and the bacilli of tuberculosis. Tuberculosis is rarely seen in horses. I find a great analogy between them.

The President: There being no further discussion, a motion to adjourn for dinner will be in order. On motion, seconded, adjourned until 1:30 P. M.

AFTERNOON SESSION.

Discussion on tuberculosis resumed. Prof. Law: In very advanced cases cattle are saturated with the poison. A drop, or a drop and a half, of tuberculin injected by syringe into the tissue, is the best diagnostic agent. You must take the temperature before using it—the day before would be better. After eight hours you may expect some rise in temperature.

Dr. French: I had a case in the city of Rome. I examined three or four head of cattle, at the request of the Board of Health. I diagnosed the disease as tuber culosis, and recommended that the animals be destroyed. The Mayor wrote to the State Board of Health at Albany. They replied, as did the Governor, but there was no action.

Prof. Law: I think we have a duty to fulfil to the community in this matter, and should respect it. The tubercles are in the muscles—go with the muscles. If we could boil them for an hour or two, protection would be satisfactory. The President: Is there anything further on this question? If not, I will appoint the following Auditing Committee: Drs. Chase, Bell and Huff.

Discussion was taken up upon the report of the Committee on Legislation.

Prof. Law: I would like to know if any one has a copy of the present law, or can recall it? The President: I think legal advice should be taken. A question comes to my mind: If men have the right to practice, how can you take it from them? What's the use of going to the Legislature until we know the value of the bill? It might prove to be unconstitutional. I propose that the Secretary be authorized to inquire into the subject. Dr. Bell: We might appoint a committee of one to act. The President: I suggest that the Secretary be instructed to take legal advice in regard to the subject, and that the committee be continued. Motion made, seconded and ordered.

Dr. Carnrite: I would like to know what was done with the bill last year, I have only heard that the bill didn't pass. A Member: It failed for want of money, as I understood. One of the members said if he was given a check for $500 he would see what he could do for it. Prof. Law: My local representative in the Legislature pledged himself to push this thing through. Each member of this Society should see his member. There is much in legislating for ourselves. Dr. Drinkwater: I think the defeat of the bill was caused by the country members. Their influence was hostile. Dr. Baker: I endorse what Prof. Law has said. If each owner would try and ascertain what he could do, the owners would have a better standing. If they would try as hard as the quacks they would be successful. We must get together and act together.

Secretary Hinkley: If you will look at the votes in the Assembly, you will find we lost our cause by a small majority.

Dr. Carnrite: I would like to make a motion that each member use his influence with the representatives from his district in the assembly, to urge the passage of this bill. Dr. Cowie: I second the motion. Being put, was declared carried.

The President: The previous motion was that the Secretary should take legal advice. He to report or not? The committee should carefully study the text of any bill to be presented. The value of the present law should be carefully analyzed. We now have a law that prevents new men entering. We cannot take the title away from those already entered. A non-graduate is a recognized practitioner; to-day it simply requires a practitioner to have been five (5) years in practice. The new law cannot act on the men already registered. You cannot take away their rights. Dr. Wende: I don't think it would deprive them of their rights. We are sitting here with our hands down—not doing anything. In my own county there a dozen practitioners liable to prosecution. I think we should have the Board of Censors or a Committee to act upon these cases.

Dr. James: I do not see how you can take away the right to practice from unregistered practitioners. The law, as I understand it, is that any one who has practiced continuously for three years can go ahead and register.

Dr. James: It is moved that a Committee of three (3) be appointed to prosecute cases of illegal registration.

Dr. Wende: Are not the County Clerks personally liable for improper registrations? Dr. Baker: I think the question was brought up at our last meeting. The officers should be authorized to prosecute unlicensed practitioners. The President: I think the Committee should be authorized to use a certain amount of money. There should be one or two test cases of prosecution. Dr. Bell: Suppose we make one test case and report at the next meeting? The President: I think that might be left to the discretion of the Committee. The motion binds the Committee without giving them any advance of money. Prof. Law: It is very important that the Committee should have funds.

Dr. Wende: I think the bill is not to "wipe out" the non-practitioners, but simply to prevent the announcement of themselves as veterinary surgeons. Prof. Law: As I understand, this committee is simply to present the bill.

Dr. Williams: I took some legal advice and found that the man who was liable was the illegal practitioner—the same as a man for false registration at the polls.

The President: I shall appoint the Secretary the Chairman of the Prosecuting Committee.

The President: At the request of the Treasurer we will take a recess for five minutes. Recess.

The Committee on By-Laws then presented a report, with a number of amendments and alterations of the existing code.

The President: Now, as to the report of the Committee on By-Laws, which were read in full by the Chairman. What is your pleasure, gentlemen, on this? If there is no objection, the Secretary will read one section at a time. Dr. Baker: I move we adopt them as a whole. The President: Gentlemen, do you wish to accept or reject?

Dr. Baker: I would like to say there should be a Financial and Recording Treasurer. They could act together for business. It would divide the burden. The President: Do you wish to do away with the Treasurer? Dr. Baker: Yes, sir; the Financial Secretary would take the place. Dr. Baker: As I understand, it is the duty of the Secretary to collect the dues, and hand them over to the Treasurer. Dr. Hinkley: I send my orders to the Treasurer, but, unfortunately, the officers are not together. They should be near to each other. Dr. Baker: I would move to amend that the Secretary should take care of the finances.

The President: The matter of the amendment of the by laws is a matter of such importance that, in my opinion, it should be laid over.

Dr. Bell: I move that the amendments be laid over until the next meeting of the Society. Seconded and carried.

The President: The next order of business is the election of officers.

On motion, seconded, the Secretary was directed to cast the ballot of the Society for Dr. Huidekoper for President for the following year. It was carried, and so declared.

The President: I thank you, gentlemen, and highly appreciate the compliment. Names are now in order for Vice-President. There were named: Drs. Wende, Bell, Drinkwater. And the ballot resulted: Drs. Wende 8, Bell 3, Drinkwater 2. Dr. Wende was, therefore, declared elected as Vice President. On motion of Dr. Baker, the election was made unanimous.

The President: The next in order is the election of a Treasurer. The nominations were: Drs. Sutterby, Baker and Bell. The vote resulted: Drs. Sutterby 4, Baker 4, Bell 8. Dr. Bell was, therefore, declared elected. On motion, the election was made unanimous.

It was declared, unanimously, that "the present worthy Secretary" should be re-elected. By motion, the President was to cast the ballot. Secretary Hinkley was declared re-elected unanimously, with great applause.

The President: The next is the election of the Board of Censors. There were placed in nomination: Drs. Sutterby, Cowie, French, Drinkwater, James, Williams, Chase.

It was declared that Drs. Chase, Drinkwater, Cowie, James and Williams were elected.

Dr. Bell: I move, Mr. President, that the Secretary read the names of those members who are in arrears for 1891. It was seconded and carried. The Secretary: Two of them have moved out of the State.

Report of the Committee on the Death of Daniel Leary, of Medina, N. Y.:

*Whereas*, It has pleased an Almighty Providence to remove from our midst a worthy member of this Society, Daniel Leary, of Medina, N. Y., be it

*Resolved*, We feel the loss of our esteemed brother and colleague, and out of due respect place on record this resolution, and extend to his bereaved family our sincere sympathy; and that a copy of the same be sent to his family, and placed on file by the Secretary of the Association.

DR. J. M. CHASE.
L. E. WILLYOUNG. } *Committee.*
JOHN WENDE.

On motion, duly seconded, the resolutions were unanimously adopted.

The following resolutions were offered by Dr. Willyoung in regard to the National Veterinary College:

No doubt the members of this Society have all noted the announcement and opening of a new veterinary school, termed the National Veterinary College, of Washington, D. C. Its Faculty is composed mostly of members and officers of the Bureau of Animal Industry; in fact, the Dean of the Faculty is the Chief of the Bureau. It is an effort (which will no doubt succeed) to establish another short term school of five and one-half months each year. We note that already nearly every State in the Union has one or more of these educational institutions, which offer great inducements to our young men, making the enterprise lucrative (even though the tuition is small) by graduating them after eleven months' study, consequently the attendance swells each year, quantity and not quality being their aim, d it is thus that sometimes our profession is brought into disrepute.

The National Veterinary College offers to its student:

1. The great public Libraries of Washington.
2. The Libraries of the Bureau of Animal Industry.
3. The National Museums. Advantages possessed by no other college.

In their introduction they say: "The courses have been arranged to suit modern requirements of Veterinary Education." Leaving it to the judgment of the profession at large, is this true? Has it not been the desire and aim of all honest co-operative veterinarians to have the courses of collegiate study lengthened, which is so necessary to elevate the standard of proficiency? Another inducement is the special feature of the school designed to fit their men for many lucrative and responsible positions, opened up by State and National legislation; a so called training school, preparing condidates for political government positions. This is practical civil service reform. Can men be prepared both practically and scientifically, in its short period of (11) eleven months?

If we have a "National school," let it be National in every sense of the word. Let it be established by the National Government, supported by the citizens of the United States, and controlled by the Government of the United States. Let us not sanction a school started and controlled entirely by servants of the United States, using the Government and its buildings, its Laboratories and its apparatus as tools to establish and maintain a private enterprise.

*Resolved*, That we, the Empire State Veterinary Medical Society, believe the establishment of another two year course school, tends to retard and menace the progress of Veterinary science in the United States. Be it further

*Resolved*, That it is the aim of the profession to elevate the standard of education, by extending the periods of study to three or four years, instead of two short courses of four to five months each. Further

*Resolved*, That we find too many young men in the profession, as in many others, who upon receiving their Diplomas, are thrown upon the world mentally unqualified and unpractical, who become discouraged, throwing up their profession, simply for want of sufficient instruction and education. Be it further

*Resolved*, That this Society appeal to the Hon. J. M. Rusk, Secretary of Agriculture, to exert his influence and power to prohibit this enterprise, which seems to us too intimately connected with the Bureau of Animal Industry.

*Resolved*, That a copy of these resolutions be sent to the Secretary of Agriculture, to the Chief of the Bureau, and to the Veterinary Journals.

L. E. WILLYOUNG, D. V. S., Albion, N. Y.

On motion, seconded, the report was adopted with the thanks of the Association.

R. S. Huidekoper, M.D., President, offered the following:

*Resolved*, That the Officers, Board of Censors and Committee of Arrangements be instructed to consult with the Comitia Minora of the United States Veterinary Medical Association, and be empowered to act in such matters as shall make the Semi-annual meeting next Summer aid in the general success of this and all other Veterinary Associations.

On motion, duly seconded, the resolution was unanimously adopted.

TREASURER'S REPORT, JANUARY 12TH, 1893.

RESOURCES:—

| | |
|---|---|
| Cash on hand | $70.62 |
| Amount due from members for dues, 1893, | 148.00 |
| " " " " " " 90–91–92, | 80.00 |
| " " " " " Initiation Fees, | 15.00 |
| " " " " " Assessments, | 138.50 |
| | $452.12 |

LIABILITIES:—

| | |
|---|---|
| Amount due to Hausman & Schmeigert, | $46.00 |
| " " " R. A. McLean, | 10.50 |
| " " " L. E. Willyoung, | 14.40 |
| " " " H. Wulff, | 12.50 |
| " " " W. B. Drake, | 75.00 |
| " " " C. D. Morris, | 50.00 |
| | $208.40 |

JOHN A. BELL,
J. M. CHASE,
WILSON HUFF.

| | |
|---|---|
| Resources in excess of Liabilities, | $243,72 |
| Liabilities in excess of Cash on hand, | 137.78 |

The Society then adjourned, subject to the call of the President.

So ordered.

N. P. HINKLEY, *Secretary.*

---

